# Sycosis

**Published:** 1888-04

**Authors:** J. T. Kent

**Affiliations:** St. Louis


### SYCOSIS.

(Notes from an extemporaneous lecture by Prof. J. T. Kent, M. D., St. Louis.)

In the further study of the miasms we now take up sycosis, which is named from one of its symptoms, a disposition to throw out figwarts.

You may wonder why you have heard so little about sycosis. The fact is that little is generally known about this miasm. Hahnemann and Bœnninghausen started the subject; its further discussion must go on, and the subject will finally be developed.

You will now and then see or hear a remark that indicates that some modern physician has seen a shadow, but the real object has seldom been observed. With one exception, as far as I know, in old-school medicine, there exists a complete darkness. This exception is Dr. Noeggerath, who thinks that latent gonorrhœa may be communicated to a woman by her husband, who has been cured (?) of this disease, which, in his opinion, is never eradicated from the system. It is the source of continual malaise, frequently the cause of early death, and often produces sterility. Diseases consequent on this are acute and chronic perimetritis, metritis, oophoritis, etc. If impregnation occurs abortion follows, or only one child is born to that woman; exceptionally, two or three. Of eighty-one women, thirty-one became pregnant and only twenty-three went to full time. Without knowing it he has corroborated the doctrine of Hahnemann and his followers. Bœnninghausen laid the foundation.

You naturally ask, How have we found out anything about sycosis, and how we know that there is such a miasm?

Out of a large volume of cases that I have gathered together, I can give but a few because of our limited time. I will mention from memory.

Years ago a man came to me with a sickly, greenish-gray countenance—a countenance even more appalling than that found in chlorosis. He had enlarged glands in the groin, he had lost much in weight, he was stiff in the joints, and the soles of his feet were very sore. Years before he had had gonorrhœa, which had been treated with injections, and the discharge had disappeared. Since then he had been taking tonics, but with no effect.

This was my first really recognized case of gonorrhœal rheumatism. I commenced to read upon the subject, but my reading was very unsatisfactory. I could find but little information. It is not necessary to say that I did not succeed in that case. That man stood before me as he stands to-day. I saw him again on crutches. His face, which stamped the picture of the miasm indelibly on my memory, still haunts me. Sickly in extreme, I see him now as he walked in and out of my office with my vague advice and prescriptions. His sallow skin and stooping frame, his hollow, wandering eyes, pleading for help, and of me, who professed to be a physician. To be sure, he had sinned, and hence the contagion, but what of his sin in comparison to that of the ignorant man who suppressed his discharge, and of the profession that fosters such ignorance, bigotry, and unbelief, in all of which we see elements that retard investigation and honest thinking?

The next case that stands up before me is that of a young baker. He had been obliged to give up his business and go to the hospital. When he was sick eighteen months, unable to work, his former employer came and asked me to do something for the poor, forsaken young man. I found him walking on his knees. The soles of his feet were so sore that he could not stand on them. His hip-joints were stiff, he was full of rheumatic symptoms, and broken down completely. These symptoms had all come on after the suppression of a gonorrhœa. I cured that young man. Some time after he was put under homœopathic treatment his gonorrhœa came back; and when the gonorrhœa was cured he had no further trouble.

A man with a most troublesome nasal catarrh, that had existed with increasing violence for eleven years, received Calcarea very high, and was converted to Homœopathy by the rapid cure of the catarrhal discharge, but it was not more than a month later that he reported that he had a discharge from the urethra, declaring that he had not been exposed to infection. He admitted that twelve years ago he had suffered from a gonorrhœa, but supposed that it was cured, as very strong injections had been used.

Dr. Wesselhœft reports an exceedingly interesting case of a man who had been troubled with vertigo for six years. Dr. Wesselhœft prescribed for him, and the vertigo disappeared, but behold! a gonorrhœa which had been suppressed for many years reappeared on the patient.

These and similar cases which I could relate give us some understanding of the beginning of that gigantic miasm which we call sycosis.

Let us start out by saying that sycosis is a constitutional and contagious disease, which sometimes, though not always, is manifested in the beginning by gonorrhœa.

There are two especial kinds of urethritic discharges. One is sycosis and the other is not. They seem alike, but you may abuse the one and not produce sycosis, while just so sure as you stop the other without curing, you have the constitutional miasm. It seems that the only relief that nature has is from the discharge, and just as soon as the discharge is stopped trouble begins.

Rheumatism is only the first shadow of the miasm, which is often observed as soon as the discharge is suppressed, but sometimes not for months after. One of my cases, where suppressed discharge was instantly followed by pains in the back and sciatics, was a great sufferer. This man writhed with double sciatics and neuralgic pains, rending in character, all over him. Constant motion relieved his pains; quiet was impossible. For many days he suffered before I found his remedy. This kind of rheumatic neuralgia is seldom attended with much swelling, the pain seems to be drawing and rending, and seems to belong largely to the nerve sheaths and tendons. Figwarts and bleeding excrescences are particularly characteristic of its later expressions. I have cured gonorrhœal rheumatism and have seen no figwarts, but in other conditions further advanced I have seen figwarts.

A man came to me with asthma. I gave him remedies which seemed to help him for a time, but I could not cure him. For a whole year I worked hard at that case. I knew that he had had gonorrhœa, but saw no relation between the gonorrhœa and the asthma. I did not understand the nature of the disease at that time. Finally I prescribed Natrum sulph. because it seemed suited to his symptoms. It wiped out the asthma completely, but in a short time figwarts began to appear about the genitals. Experience has shown that whenever these figwarts are burned off, deep-seated constitutional diseases invariably follow. I did not burn them, I gave him Thuja, which is complementary to Natrum sulph., and suited to the case. The figwarts disappeared and his old gonorrhœa discharge came back, which was, as these cases usually are, most difficult to cure.

The returning discharge is often unmanageable, and may resist treatment for years, because the miasm has become deep seated, and the discharge should exist until this miasm has been cured. Treating the discharge as a gleet is a most dangerous and unhomœopathic management.

Can you see anything in these facts in the light of what I have taught you, and do you see the relation of all these facts to each other?

Had I stopped that discharge by suppression, his old asthma would have come back. I would not have known why, and no one would have blamed me. Many such things have I seen. I practiced that way before I knew better, and I know whereof I speak. I have related to you some points that show you how I first saw a glimmering of the truth, but the great field is yet to be explored by you.

The manifestations of sycosis are often much like those of syphilis and psora, when each is in its latency or suppressed. You have aches and pains in the beginning of all three of the miasms, that resemble each other very much. Later, after the results of the disease have become evident through tissue changes, each miasm stands out in bold relief.

In syphilis, when its surface eruptions are driven back, it finally attacks the nerve centres, bone cells, and periosteum. Psora is more general in its nature. It attacks the skin and all parts of the body.

To-day I believe that sycosis is as deep a miasm as syphilis, with just as destructive a blood disorganization; therefore the anæmic aspect, waxy, greasy skin, red, smooth warts on mucous margins of anus and genitals, loose teeth, extreme nervous tension, phthisical condition, catarrhs wherever there are mucous membranes, epithelioma, and emaciation.

Some one says that if sycosis is so deep a miasm, would it not be a good thing to take the gonorrhœal virus and prove its effect upon the human system, in order to bring out the disease where we can study it? This has been done.

Medorrhinum is such a substance, and students who have access to my office know that I have quite a volume of provings of Medorrhinum through the favor of Dr. Swan. It would seem that provers had nearly sacrificed their lives to bring out the action of this miasm.

This proving of Medorrhinum brings out the rheumatic states, the soreness in the bottoms of the feet, headache in day-time, periodical headaches, restlessness, pains from sunrise to sunset, which are so characteristic of sycosis (syphilis has pains at night, from sunset to sunrise), and many deeper symptoms which are found in sycotic diseases. This proving confirms everything that I have told you about sycosis—all that we learn from the study of the disease itself.

Many severe cases of asthma, the result of suppressed gonorrhœa, are speedily cured by Medorrhinum and the symptoms of sycosis are brought out. Medorrhinum develops the suppressed miasm, so that its symptoms are harmonious and consistent. It does not cure the miasm. It does not cure gonorrhœa. It acts as a developing remedy, as does Psorinum and Syphilinum in the other miasms.

Deep rheumatic attacks are often due to gonorrhœa, though this is not always recognized.

Children may be born sycotic, where one or both parents are afflicted with gonorrhœa. Such children are likely to have cholera infantum, marasmus—pining children. I have watched these cases and have often found Medorrhinum the only medicine which will save the lives of these little ones.

As Psorinum has many times brought about a vital reaction after a typhoid fever when all energies were suspended, and when psora was at the bottom of the trouble, so will Syphilinum cause the same vital reaction if it be syphilis that is the cause of the suspended energy when convalescence is prevented; and so also will Medorrhinum cause a reaction when the sycotic miasm is the cause of slow convalescence. A careful study of the provings and ample clinical experience lead me to state these things with assurance.

Psorinum does not cure psora, and Syphilinum does not cure syphilis, nor does Medorrhinum cure sycosis.

I have traced epithelioma, red phthisis, cauliflower excrescences, sterility, and erosions to a sycotic origin. Pernicious anæmia often has gonorrhœa as its base.

This led me to the discovery of Picric acid as a sycotic medicine through its relations to pernicious anæmia. It even cures figwarts and gonorrhœa—of course, when indicated.

Iritis is supposed to belong almost exclusively to syphilis; syphilis when not suppressed may produce iritis. Gonorrhœa produces it only when suppressed.

One, two, or all three miasms may exist in the system at the same time. They may complicate each other. Let a patient start out with psora, then let syphilis ravage his system, finally the gonorrhœal miasm is added, while all the time he is being filled and overpowered with drugs.

Just think what a complication we have to deal with.

Hahnemann recognizes the alternation of one miasm with another. He gives Merc. for syphilis, perhaps then psora comes uppermost and he finds Sulph. indicated, then sycosis comes and alternates with one or the other, and so on.

Make note of what you see in the backward course of disease (*i. e.*, when it is getting well), and you will see more and more the relation of these things. Do not make haste to prescribe for old symptoms that come back. Be sure that the symptom is going to stay, for you will have flitting images. Old symptoms come and go and need no further repetition of the medicine.

If you give a new remedy when not needed, you spoil your case. Never prescribe for a moving image, wait till it rests. 'Tis your duty to understand your business before you attempt to do anything.

Miasms are the foundation of all chronic diseases. He who sees in Bright's disease nothing but Bright's disease, not the deep miasm back of it, sees not the whole disease but only the finishing of a long course of symptoms which have been developing for many years.

If you go at it like a common tinker you may cure acute sickness, but on your life, do not tamper with these chronic diseases. With your best endeavor you will make mistakes, but make them as few as you can. Do you see the necessity of going to the foundation of these things?

